# A general approach for postmortem interval based on uniformly distributed and interconnected qualitative indicators

**DOI:** 10.1007/s00414-016-1520-3

**Published:** 2017-01-04

**Authors:** Szymon Matuszewski

**Affiliations:** Laboratory of Criminalistics, Adam Mickiewicz University, Św. Marcin 90, 61-809 Poznań, Poland

**Keywords:** Forensic science, Postmortem interval, Carrion insects, Succession

## Abstract

There are many qualitative indicators for postmortem interval (PMI) of human or animal cadavers. When such indicators are uniformly spaced over PMI, the resultant distribution may be very useful for the estimation of PMI. Existing methods of estimation rely on indicator persistence time that is, however, difficult to estimate because of its dependence on many interacting factors, of which forensic scientists are usually unaware in casework. In this article, an approach is developed for the estimation of PMI from qualitative markers in which indicator persistence time is not used. The method involves the estimation of an interval preceding appearance of a marker on cadaver called the pre-appearance interval (PAI). PMI is delineated by PAI for two consecutive markers: the one being recorded on the cadaver (defining lower PMI) and the other that is next along the PMI timeline but yet absent on the cadaver (defining upper PMI). The approach was calibrated for use with subsequent life stages of carrion insects and tested using results of pig cadaver experiments. Results demonstrate that the presence and absence of the subsequent developmental stages of carrion insects, coupled with the estimation of their PAI, gives a reliable and easily accessible knowledge of PMI in a forensic context.

**Electronic supplementary material:**

The online version of this article (doi:10.1007/s00414-016-1520-3) contains supplementary material, which is available to authorized users.

## Introduction

### Background

Forensic scientists have identified several postmortem interval (PMI) indicators and developed many technical methods for PMI estimation [1]. Although there are good quantitative indicators, for example, cadaver temperature [2], potassium content in vitreous humor [3], or the length of carrion insect larvae [4, 5], many markers are qualitative, for example, species/life stages of insects or bacteria successively occurring on cadavers [6–9] or markers related to soft tissue decomposition [10, 11]. As a rule, qualitative markers persist for some time, and the persistence times of different markers usually largely overlap. However, when such indicators are uniformly distributed over PMI [12], resultant distribution may be particularly useful for the estimation of PMI. Regular distributions were found for larval insects successively appearing on cadavers [7, 13], developmental landmarks of carrion insects [14–16], or indicators related to soft tissue decay [10, 11].

**Fig. 1:**
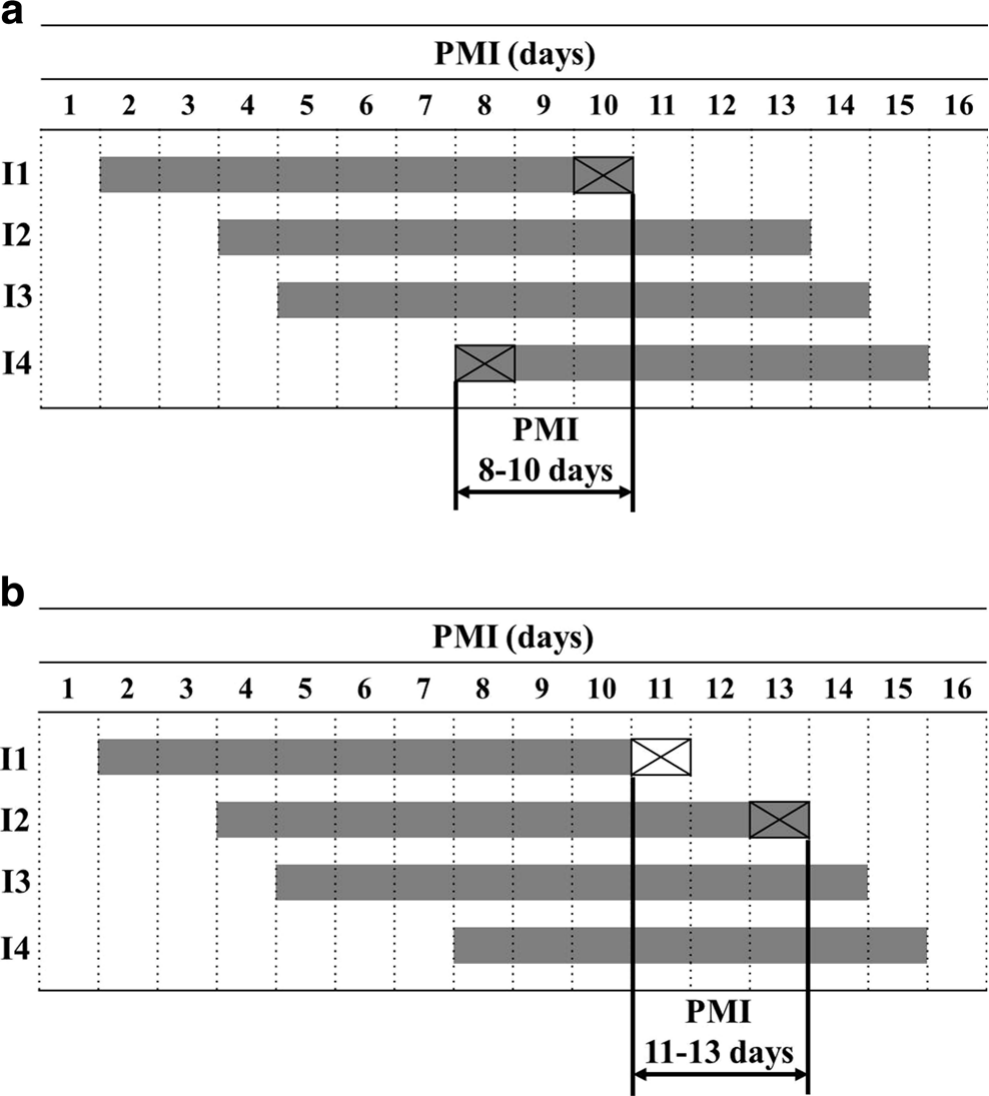
A schematic representation of methods for PMI estimation from qualitative indicators. **a** The “indicator presence” method. **b** The “indicator absence/presence” method. *I1*, *I2*, *I3*, *I4* indicators no. 1, 2, 3, and 4

Based on qualitative indicators, an approach for PMI was developed by Schoenly et al. for insect successional markers [17, 18]. A similar method was proposed for developmental indicators of blowfly pupae [14] and immunohistochemical markers [19]; it is called the “indicator presence” method, as an estimate is based on the presence of two markers: the one that starts later and the other that ends earlier compared to the other markers recorded (Fig. 1a). The former defines lower PMI, the latter upper PMI. Another approach, applied by forensic entomologists [20], is called the “indicator absence/presence” method (Fig. 1b). It involves two markers: lower PMI is, however, defined by the absence of an early indicator and only upper PMI is delineated by the presence of a long-lasting indicator (Fig. 1b). Both methods rely on indicator persistence time (IPT), delineating upper PMI for the first method or both lower and upper PMI in the case of the second (Fig. 1a, b). IPTs are, however, difficult to estimate due to their large variation. The rate and duration of cadaver decomposition are influenced by many factors, for example, the type of cadaver exposure [21–23], temperature [10], access by insects [24, 25], or cadaver mass [26, 27]. Persistence of indicators directly or indirectly related to decomposition (e.g., carrion insects or bacteria) is similarly affected. Carrion insects, for example, persist longer on large cadavers [26] or in colder seasons [7, 28]. Accordingly, the reliance on IPTs reduces accuracy of existing methods. In this article, an approach is developed for the estimation of PMI from qualitative indicators, in which IPTs are not used.

### An outline of the method

**Fig. 2:**
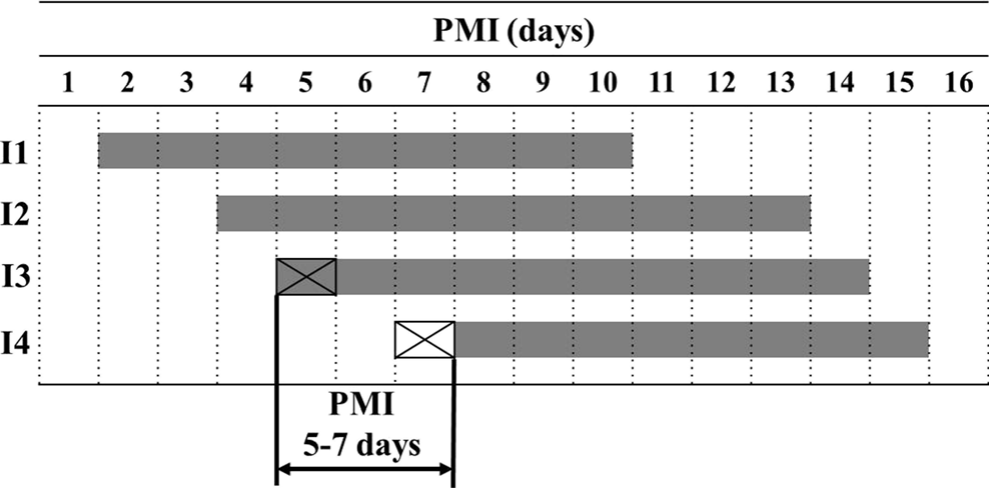
A schematic representation of the “indicator presence/absence” method. *I1*, *I2*, *I3*, *I4* indicators no. 1, 2, 3. and 4

The approach is based on appearance times of subsequent and interconnected indicators. It uses two indicators and involves the estimation of an interval preceding their appearance on a cadaver (hereafter referred as a pre-appearance interval, PAI). Reliance of the method on PAI is its advantage, as it was demonstrated that PAI for some indicators is unrelated to cadaver mass (e.g., insects [26]) and may easily be estimated using temperature methods [29]. Lower PMI is delineated by PAI of the present indicator and upper PMI by PAI of the indicator that is next along the PMI timeline but yet absent on the cadaver (Fig. 2). A similar logic of estimation was proposed by Reh for the estimation of post-submersion interval from morphological indicators of immersed cadavers [30, 31]. Although decomposition charts of Reh were established for the estimation of minimum PMI, it was suggested that maximum PMI may be estimated as well, by analyzing the indicators that have not yet developed in the cadaver [31]. The compound method for the early PMI estimation of Henssge et al. [32, 33] incorporates similar mechanics of estimation.

**Fig. 3:**
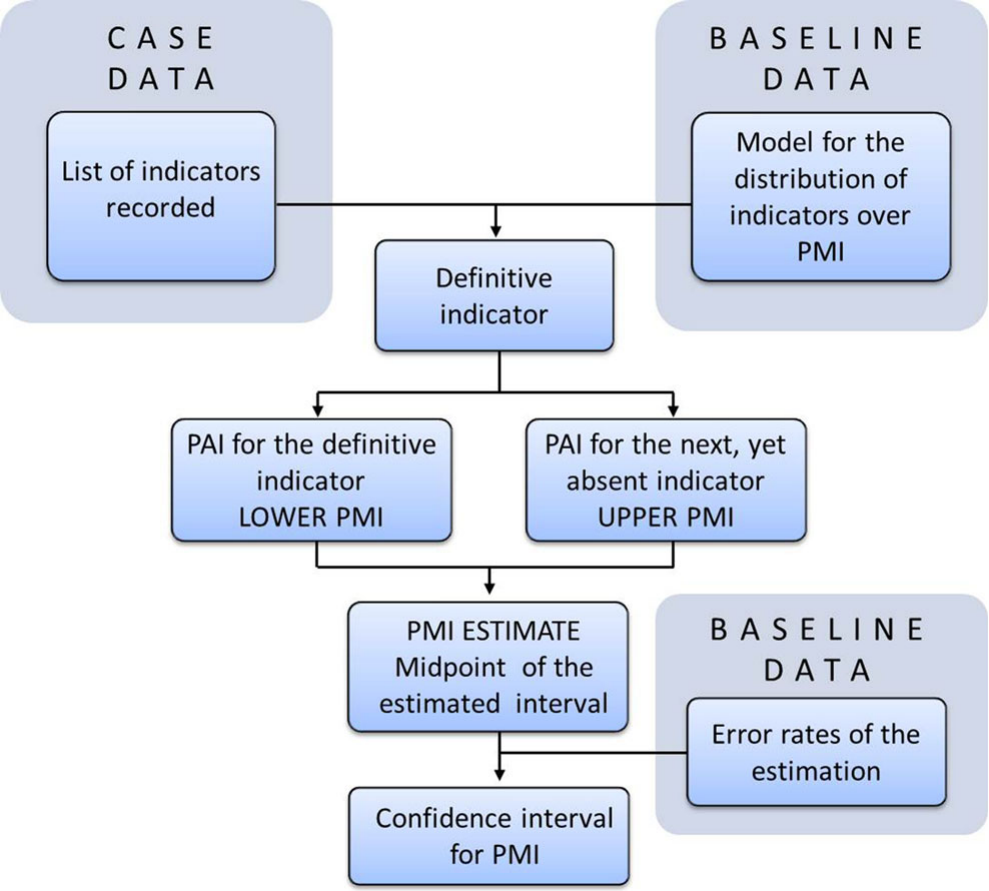
The procedure for PMI estimation from uniformly distributed and interconnected qualitative indicators

The method may be divided into several steps (Fig. 3). First, cadaver is examined and relevant indicators are documented. Second, the definitive indicator (i.e., the one that starts later than the others) is chosen through comparing indicators revealed on the cadaver against the baseline distribution of indicators. The third step involves the estimation of PAI for the definitive indicator, and the fourth step consists of the estimation of PAI for the subsequent yet absent indicator. The sequence in which indicators occur on a cadaver should be stable. Moreover, indicators should occur with high regularity irrespective of case circumstances. These requirements are met by interconnected indicators, that is, markers inherently related to each other as, for example, subsequent life stages of carrion insects. An interval delineated by lower and upper PMI (i.e., the estimated interval) may be very narrow, and for this reason, it was assumed that the true PMI may regularly lie outside of this interval. Consequently, taking the midpoint between the lower and upper PMI, the method involves transforming this interval into the point estimate. The final result is presented as an interval around this point estimate and is generated using previously specified error rates of the method. Because the procedure involves several sources of error (e.g., indicator documentation error, PAI estimation error, upper-lower PMI error, etc.), it is assumed that these conversions allow to provide highly informative and robust interval estimate by incorporating the single error rate of the whole method.

### Calibration of the method for insect evidence

Carrion insects have long been recognized as useful in estimating PMI [34]. Several insect-related processes were tested or used for this purpose [35, 36], and the ecological succession was presumably the one focusing largest scientific interest [34]. The succession of insect life stages on carrion represents a regular pattern; therefore, life stages of carrion insects will be used as indicators and their PAI as delineation for lower and upper PMI.

The method’s several components may be distinguished (Online Resource: ESM 1). First, corpse fauna is collected, identified, and classified. Second, a definitive species (i.e., the one that colonizes cadavers later than the others) and stage (i.e., the most developmentally advanced life stage of the definitive species) are chosen. In the third step, PAI of the definitive life stage is estimated. When PAI of a stage is closely related to the preceding temperature, it may be estimated using case-specific temperature data and temperature model for PAI [29]; when it is poorly related to the preceding temperature, an average seasonal or monthly PAI may be more useful [29, 37]. In the fourth step, PAI of the next yet absent stage is estimated using the same methods and data as in the previous step. For example, if corpse fauna comprises 1st and 2nd instar larvae of *Necrodes littoralis* (Coleoptera: Silphidae) and 3rd instar larvae of *Lucilia caesar* (Diptera: Calliphoridae), 2nd instar larvae of *N. littoralis* should be considered as definitive because *N. littoralis* colonizes cadavers later than *L. caesar* and its 2nd instar larvae are the most developmentally advanced. Consequently, PAI of the 2nd larval stage of *N. littoralis* defines lower PMI; PAI of the next yet absent 3rd larval stage of *N. littoralis* defines the upper PMI. In the last step, using the error rate of the method, the midpoint between lower and upper PMI is transformed into the final interval for PMI.

## Materials and methods

Based on the results of previous pig carcass studies [26, 38–40] and case histories [41–44], a list of definitive insect species was prepared for the rural habitats of Central Europe (Online Resource: ESM 2). The only precondition the species had to meet was regular breeding in large cadavers. Species to be used in the validation tests were selected based on their distribution over PMI and availability of the necessary data.

Temperature models for PAI of developmental stages were estimated using previous methods [45] and data from previous studies [46, 47]. It was assumed that PAI starts at the moment of death and ends in the midpoint between the first collection of relevant insect specimen and the time when previous sample was taken. Although oviposition PAI of most carrion flies is poorly related to preceding temperature [46], it was assumed that the dependence of PAI on temperature will get stronger for later life stages due to the strong effect of temperature on the developmental rate of insects [5]. Accordingly, temperature models for PAI were also created for life stages of flies.

The method was validated using results of previous experiment [26, 27], with pig carcasses exposed in xerothermic grasslands (Western Poland, Europe; 52°31′N, 16°55′E) during spring, early, and late summer of 2012. Each seasonal block comprised eight cadavers (naked and clothed, cadaver mass 7–64 kg). Insects were sampled manually and with pitfall traps. Ground level temperatures were recorded at every carcass. The baseline data used to validate the method were different from the data used to develop the PAI models from the previous paragraph.

The validation procedure comprised several steps. First, relevant data were extracted from insect occurrence records of the baseline experiment; they included PMIs in which relevant configurations of life stages (presence/absence of subsequent stages) had been observed (hereafter referred as true PMI). In the second step, the current method was used to estimate PMI (hereafter referred as estimated PMI). Estimates were made for each day with relevant configuration of life stages. Temperature records were obtained from a local weather station and were retrospectively corrected to accommodate systematic differences between weather station and places where cadavers were exposed [48]. Then, using the temperature method, PAI for the present developmental stage was estimated [29]. The predictor temperature (i.e., temperature used to predict PAI with the model) was approximated using the following procedure. The average monthly PAI was extracted from a local carrion insect database. Temperature was averaged for this PAI starting from the day when the given configuration had been recorded and calculating backward. Corrected weather station temperatures were used in these calculations. The resultant average temperature was used as the first approximation of predictor temperature, and eventually the first PAI estimate was made. This procedure was iterated twice because such iterations were found to improve the approximation of predictor temperature and resultant estimate of PAI [29]. While iterating, in each case, the PAI estimate from the previous iteration was used to approximate predictor temperature in the next iteration. In the end, the 3rd estimate of PAI was used as the lower PMI. Using the same method and data, PAI for the absent life stage (defining upper PMI) was estimated. The third approximation of predictor temperature as used for the PAI of the present life stage in the previous step was used for this purpose. Average monthly PAIs were used instead of temperature estimates for these life stages that reveal a poor relationship between PAI and temperature. Monthly PAIs were calculated based on the same data from which temperature models were estimated. The midpoint between resultant lower and upper PMI is the estimated PMI. Next, error rate of the method was analyzed.

All analyzes were made at the 5% level of significance using Statistica 12 (StatSoft, Inc.).

## Results

Due to the finely uniform distribution of their life stages over PMI, *L. caesar* (Diptera: Calliphoridae), *Thanatophilus sinuatus*, and *N. littoralis* (Coleoptera: Silphidae) met the requirements to be included in the tests (Online Resource: ESM 1). Larval instars of the species and additionally the egg stage and the post-feeding larval stage of *L. caesar* were used as indicators. Consequently, eight configurations were tested (Table 1), covering about 20 days of decomposition. Temperature models for PAI were of acceptable quality for the 2nd and the 3rd larval stages (feeding and post-feeding phase) of *L. caesar* and for all the larval stages of *T. sinuatus* and *N. littoralis* (Online Resource: ESM 1-2). Due to the low quality of temperature models, the average monthly PAIs were used for the egg stage and the 1st larval stage of *L. caesar* (Online Resource: ESM 2).

**Table 1:** Tested configurations of indicators

Species	Configuration of indicators	Abbreviation
*Lucilia caesar*	Presence of eggs and absence of 1st instar larvae	Eggs/1st
	Presence of 1st instar larvae and absence of 2nd instar larvae	1st/2nd
	Presence of 2nd instar larvae and absence of 3rd instar larvae	2nd/3rd
	Presence of 3rd instar larvae and absence of post-feeding larvae	3rd/post-feeding
*Thanatophilus sinuatus*	Presence of 1st instar larvae and absence of 2nd instar larvae	1st/2nd
	Presence of 2nd instar larvae and absence of 3rd instar larvae	2nd/3rd
*Necrodes littoralis*	Presence of 1st instar larvae and absence of 2nd instar larvae	1st/2nd
	Presence of 2nd instar larvae and absence of 3rd instar larvae	2nd/3rd

**Fig. 4:**
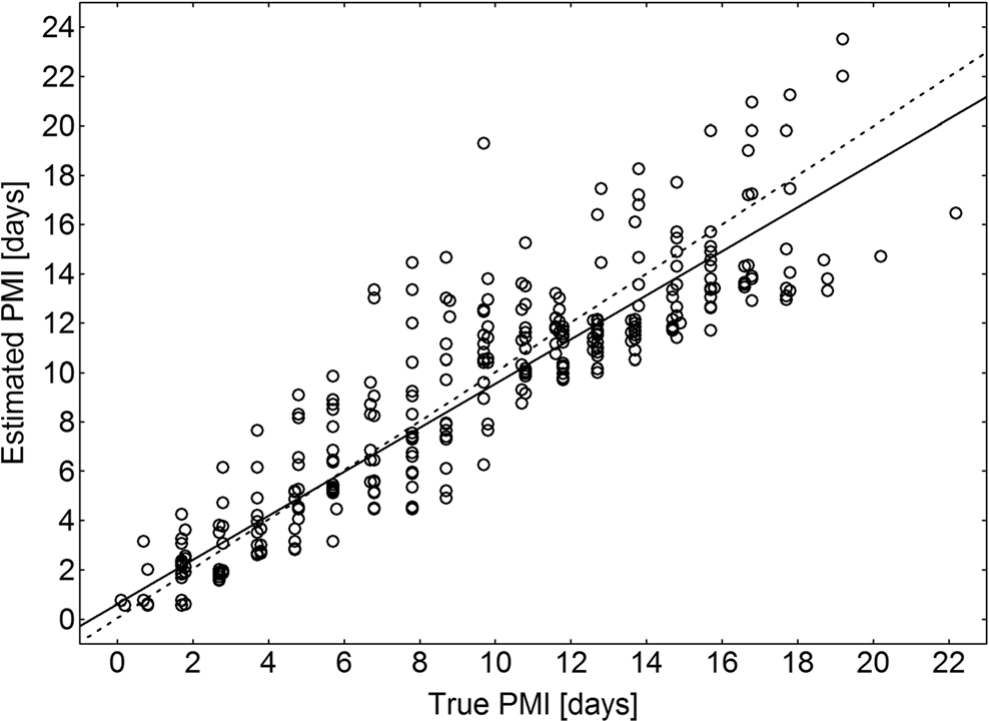
Results of PMI estimation for eight configurations of insect indicators. *Solid line*, regression model of the relationship between true and estimated PMI. *Dotted line*, hypothetical line representing perfectly accurate estimates

**Fig. 5:**
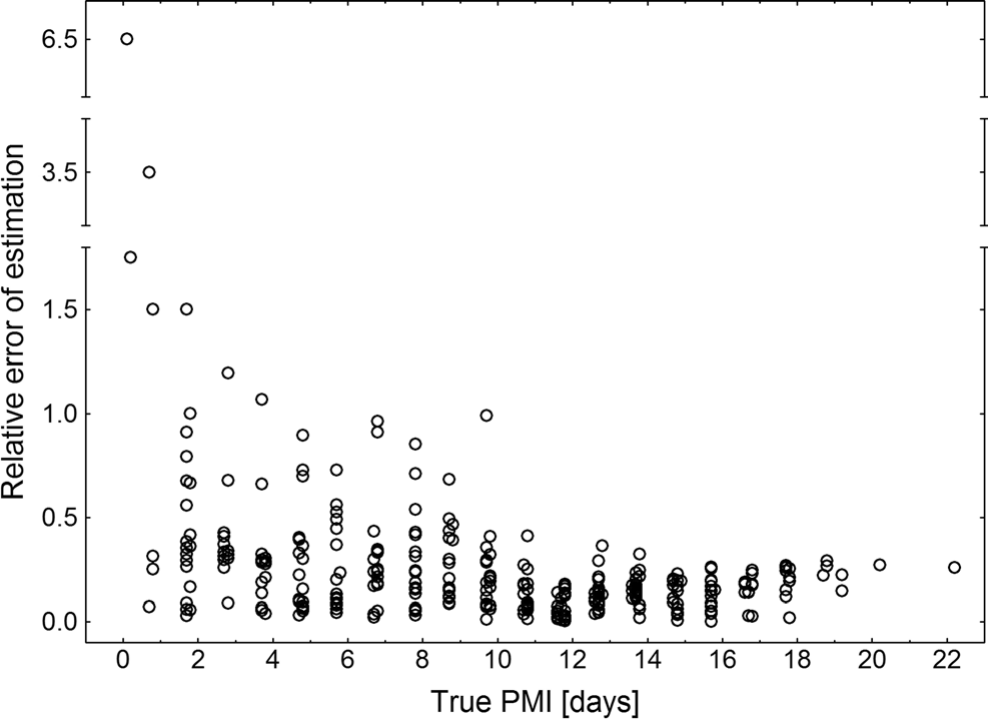
Relative error of estimation (absolute difference between true and estimated PMI divided by true PMI) plotted against true PMI

PMI estimates were highly aggregated around the line representing perfectly accurate estimates in the entire PMI range (Fig. 4). A regression model for the relationship between estimated and true PMI (linear regression, estimated PMI = 0.6173 + 0.8934 * true PMI, *t* = 42.3, *P* < 0.001, *r* ^2^ = 0.83, Fig. 4) only slightly deviated from the line representing perfect estimates (Fig. 4). Relative error of estimation decreased with an increase in PMI (Fig. 5). This finding suggests that configurations relevant for short PMI have higher estimation error than configurations relevant for long PMI. A formal comparison of configurations according to the error rate revealed highly significant differences (Kruskal-Wallis test, H_(7, 371)_ = 88.8, *P* < 0.001), with beetle configurations having lower error rates than fly configurations (Table 2, Online Resource: ESM 1). Error rates were not related to carcass mass (linear regression, relative error of estimation = 0.30057 − 0.000735 * carcass mass, *t* = −0.62, *P* = 0.535, *r* ^2^ = 0.001, Online Resource: ESM 1).

**Table 2:** Accuracy of PMI estimates with the current method

Species	Configuration of indicators	Accuracy of PMI estimation							
		N	Inclusions^a^		Mean PMI width^b^ (days)	Absolute error (days)^c^		Relative error^d^	
			N	%		Mean	Range	Mean	Range
*Lucilia caesar*	Eggs/1st	24	16	67	0.72	0.404	0.05–1.20	0.684	0–6.50
	1st/2nd	38	24	63	2.31	0.920	0.05–2.55	0.514	0.03–3.50
	2nd/3rd	37	10	27	1.59	1.201	0.15–3.35	0.327	0.04–1.20
	3rd/post-feeding	75	42	56	3.00	1.651	0.15–4.30	0.268	0.02–1.07
*Thanatophilus sinuatus*	1st/2nd	50	28	56	3.03	2.000	0.10–6.65	0.235	0.01–0.96
	2nd/3rd	52	21	40	2.24	1.647	0.05–9.60	0.142	0–0.99
*Necrodes littoralis*	1st/2nd	53	18	34	2.30	1.775	0–4.65	0.130	0–0.41
	2nd/3rd	42	2	5	2.00	3.131	0.50–5.75	0.181	0.03–0.29

*Eggs/1st* presence of eggs and absence of 1st instar larvae, *1st/2nd* presence of 1st instar larvae and absence of 2nd instar larvae, *2nd/3rd* presence of 2nd instar larvae and absence of 3rd instar larvae, *3rd/post-feeding* presence of 3rd instar larvae and absence of post-feeding larvae

^a^Cases when the true PMI lay within the estimated interval (interval between lower and upper PMI)

^b^Mean difference between upper and lower PMI (Schoenly et al. 1996)

^c^Absolute difference between true and estimated PMI (i.e., the midpoint of the estimated interval)

^d^The absolute error divided by the true PMI

Confidence intervals (Table 3) based on practical error rates (Online Resource: ESM 1) were narrower for beetle configurations than for fly configurations; they were reasonably small for beetle configurations, indicating that the method may give robust PMI estimates.

**Table 3:** Confidence limits for PMI estimates based on practical error rates

Species	Configuration of indicators	N	Confidence limits^a^		
			90%	95%	99%
*Lucilia caesar*	Eggs/1st	24	−0.64*x*;2.09*x*	−0.87*x*;2.09*x*	−0.87*x*;2.09*x*
	1st/2nd	38	−0.60*x*;0.74*x*	−0.78*x*;0.74*x*	−0.78*x*;0.74*x*
	2nd/3rd	37	−0.40*x*;0.68*x*	−0.54*x*;0.81*x*	−0.54*x*;0.81*x*
	3rd/post-feeding	75	−0.42*x*;0.71*x*	−0.47*x*;0.75*x*	−0.52*x*;0.78*x*
*Thanatophilus sinuatus*	1st/2nd	50	−0.46*x*;0.22*x*	−0.48*x*;0.24*x*	−0.49*x*;0.28*x*
	2nd/3rd	52	−0.24*x*;0.26*x*	−0.29*x*;0.27*x*	−0.50*x*;0.30*x*
*Necrodes littoralis*	1st/2nd	53	−0.24*x*;0.30*x*	−0.27*x*;0.34*x*	−0.29*x*;0.34*x*
	2nd/3rd	42	−0.18*x*;0.37*x*	−0.20*x*;0.37*x*	−0.21*x*;0.41*x*

*Eggs/1st* presence of eggs and absence of 1st instar larvae, *1st/2nd* presence of 1st instar larvae and absence of 2nd instar larvae, *2nd/3rd* presence of 2nd instar larvae and absence of 3rd instar larvae, *3rd/post-feeding* presence of 3rd instar larvae and absence of post-feeding larvae

^a^Confidence limits (lower; upper) based on practical error rates calculated for PMI estimates from this study, *practical error rate* the difference between true and estimated PMI divided by estimated PMI, *x* estimated PMI

## Discussion

Current results demonstrate that insect successional indicators may produce accurate PMI estimates without using indicator persistence time (IPT). When they are interconnected and uniformly distributed over PMI, the presence and absence of subsequent indicators (here life stages of carrion insects) coupled with the estimation of their PAI gives a reliable and easily accessible knowledge of PMI.

The method has several advantages. First, it covers a wide range of PMI, similar to development-based entomological methods [5] or decomposition-based taphonomic methods [10]. Moreover, through the inclusion of other species and life stages, this range may be expanded outperforming development-based entomological methods. *Stearibia nigriceps* (Diptera: Piophilidae), *Omosita colon* (Coleoptera: Nitidulidae), species of *Necrobia* (Coleoptera: Cleridae) or *Dermestes* (Coleoptera: Dermestidae), and parasitoids of blowfly pupae, for example, *Nasonia vitripennis* (Hymenoptera: Pteromalidae), regularly colonize cadavers long after death [7, 26, 41, 49] and, from this point of view, may expand the range until about 3 months after death. Inclusion of eggs, pupae, and tenerals may have similar effect. Second, the method has a fine resolution, dividing PMI into many uniform and narrow subintervals. From this point of view, it outperforms decomposition-based taphonomic methods, in case of which subintervals enlarge with an increase in PMI [10]. Third, the method is accurate, particularly with these configurations for which PAI may be estimated using temperature methods. Although some methods have lower error rates, for example, methods based on cadaver temperature [50], accuracy of the current method is higher than some other approaches relevant for long PMI, for example, decomposition-based taphonomic methods [10, 51], or similar as compared to the others, for example, development-based entomological methods. Fourth, it may be easily applied in the forensic routine, as it needs only good insect sample and reliable temperature data. Fifth, accuracy of PMI estimation is unrelated to cadaver mass, and, from this point of view, the method outperforms other insect successional methods [17, 18, 52].

The method has, however, also some disadvantages. First, its good performance depends on the professional sampling of insects. Several life stages used are difficult to be sampled due to their small size (eggs or first instar larvae) or cryptic behavior (e.g., larvae of some beetle species). Although these effects may be reduced by including accessory species (i.e., species colonizing cadavers at time regimes similar to the major species), without standardized and professionalized sampling on a crime scene, the method may be ineffective. Second, the method needs sophisticated baseline data, that is, temperature models for PAI or average monthly or seasonal PAIs for particular life stages of carrion insects. Current protocols for decomposition studies cannot provide such data, and consequently, rebuilding them will be necessary. A framework for novel protocol was published recently [45]. Third, problems may arise with recolonizing taxa [12], as they may have two (or more) separated intervals during which the same configuration of indicators is present. A recent study revealed that species feeding on long-lasting carrion parts or arthropods present in such parts frequently recolonize on large cadavers [40]. Moreover, it was indicated that winter break in insect activity is necessary for the occurrence of recolonization [40]. Therefore, these estimation problems may occur for overwintered cadavers, for which the method seems to be inapplicable. Another possible disadvantage is the dependence of the method upon the environment in which cadaver was found. Because environments differ in composition of carrion fauna [53–55], cadavers in different environments may have different definitive species. Moreover, when average monthly PAI is used instead of temperature estimates, the most accurate PMI estimates will be given from the PAI data specific for the environment in which cadaver was found. The questions whether average monthly or seasonal PAIs differ across environments and how large are these differences remain, however, open. From the other side, in the case of these taxa for which PAI may be estimated using temperature methods, validation studies for these methods [29, 56, 57] suggest that a single PAI model may be used across habitats. Moreover, previous data suggest that temperature models for PAI may give accurate estimates for insects from different geographic populations [56], and this finding indicates that the current PAI models may be to some extent used also across different geographic areas. All these problems need, however, further studies.

Although the method was calibrated for immature insects, it may be tempting to include adult insects. Most previous successional approaches for PMI used adult insects as primary indicators [9, 17, 52]. The inclusion of adult insects may be, however, problematic for two reasons. First, adult insects may be present on cadaver longer than all immature life stages. For this reason, the presence of adult stage and absence of 1st instar larval stage may occur twice, that is, at the beginning and at the end of adult stage residency, and this may decrease the accuracy of PMI estimation with the method. Second, there are carrion species that may be present on some cadavers exclusively as adult stage, for example, *N. littoralis* on some small or medium cadavers was found only as an adult stage [26]. In such cases, the time regime during which adult insects are present and 1st instar larval insects are absent may be distinctly prolonged, affecting the accuracy of estimation.

Although the method was tested only with insect successional markers, it may be similarly effective with other qualitative markers, in particular the ones that are reasonably interconnected and uniformly spaced over PMI. From this point of view, insect developmental indicators are very promising. Insect development may be easily represented as a sequence of qualitative changes uniformly spaced over insect life cycle and eventually over PMI. Forensic entomologists documented many examples of such stepwise distributions of external or internal morphological characters over the developmental timeline [14–16, 58]. The current logic of estimation may be used to determine insect age from such distributions. Some morphological characters of immature insects may, however, also be used as direct PMI markers, similarly to the larval instars that were used here as primary indicators. Although modeling their occurrence along the PMI timeline will be challenging, the gain in resolution will compensate the necessary research efforts. Taphonomic markers are similarly promising. Cadaver decomposition may be described as a stepwise distribution of qualitative characters [1, 10], and some of them, for example, rigor mortis, bloating, or bone exposure, nicely fit the current approach for PMI. Moreover, it seems that all qualitative markers (entomological, taphonomic, etc.) may be combined in a single, multimarker method for PMI, incorporating the logic of estimation described in this article.

## Electronic supplementary material

ESM 1 (PDF 1439 kb)

ESM 2 (PDF 214 kb)
