# Supplementary material for: A general approach for postmortem interval based on uniformly distributed and interconnected qualitative indicators
Source: Int J Legal Med. 2017 Jan 4;131(3):877–84. doi: 10.1007/s00414-016-1520-3 (PMC5388707; doi:10.1007/s00414-016-1520-3)
Supplement: Supplementary file 1 — (PDF 1439 kb) [file 414_2016_1520_MOESM1_ESM.pdf]

SUPPLEMENTARY FIGURES FOR

"A general approach for postmortem interval based on uniformly distributed and interconnected qualitative indicators"

International Journal of Legal Medicine

Szymon Matuszewski

Laboratory of Criminalistics, Adam Mickiewicz University, Św. Marcin 90, 61-809 Poznań, Poland

szymmat@amu.edu.pl.

Content

Fig. 1. Calibration of the method for insect evidence

Fig. 2. Distribution of tested insect indicators over PMI

Fig. 3. The pre-appearance interval (PAI) of *Lucilia caesar* life stages plotted against ground level temperature averaged over PAI

Fig. 4. The pre-appearance interval (PAI) of *Thanatophilus sinuatus* life stages plotted against ground level temperature averaged over PAI

Fig. 5. The pre-appearance interval (PAI) of *Necrodes littoralis* life stages plotted against ground level temperature averaged over PAI

Fig. 6. Relative error of estimation plotted against carcass mass

Fig. 7. Differences between configurations in the relative error of estimation

Fig. 8. Practical error rates for particular configurations

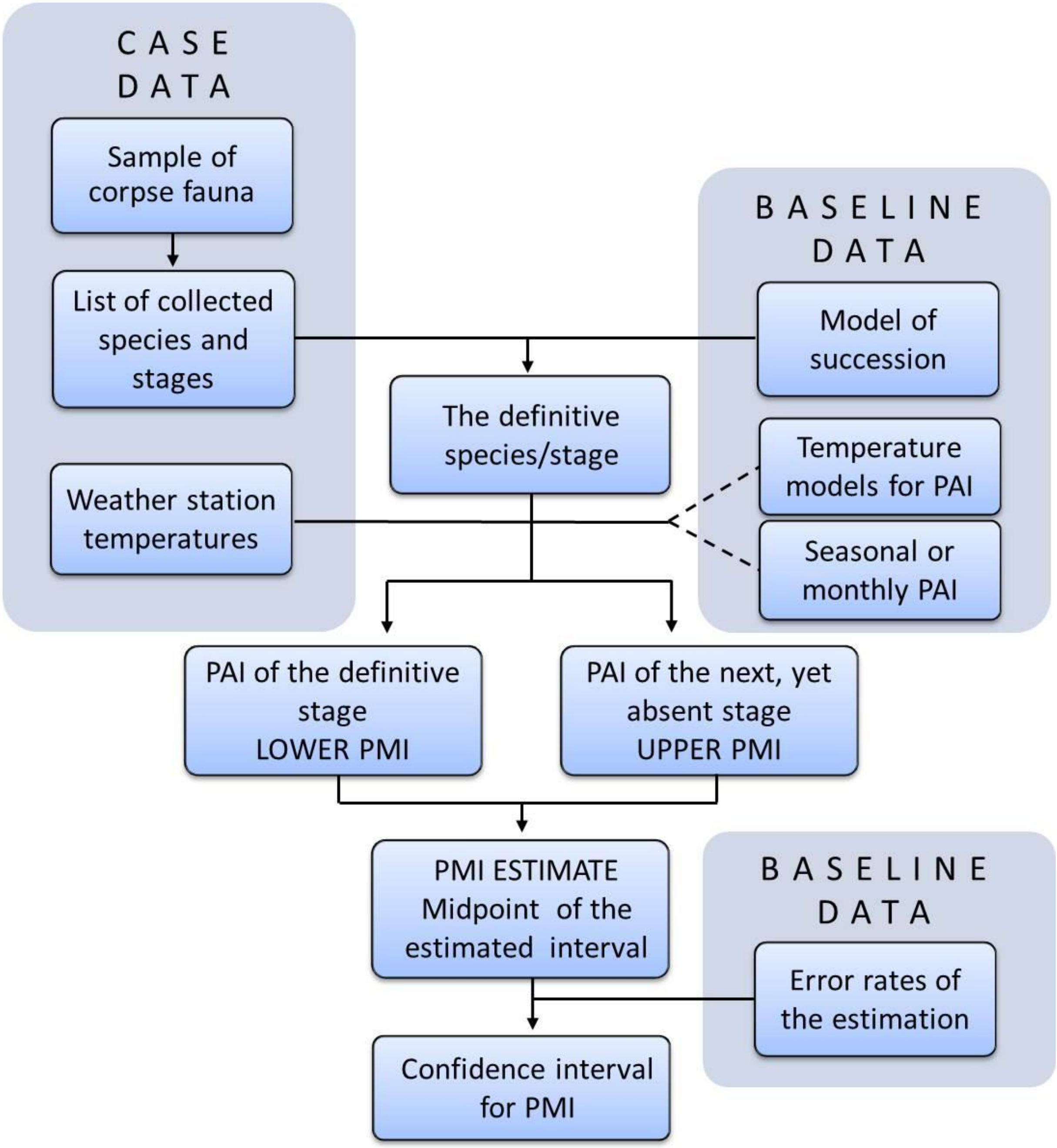

Fig. 1. Calibration of the method for insect evidence.

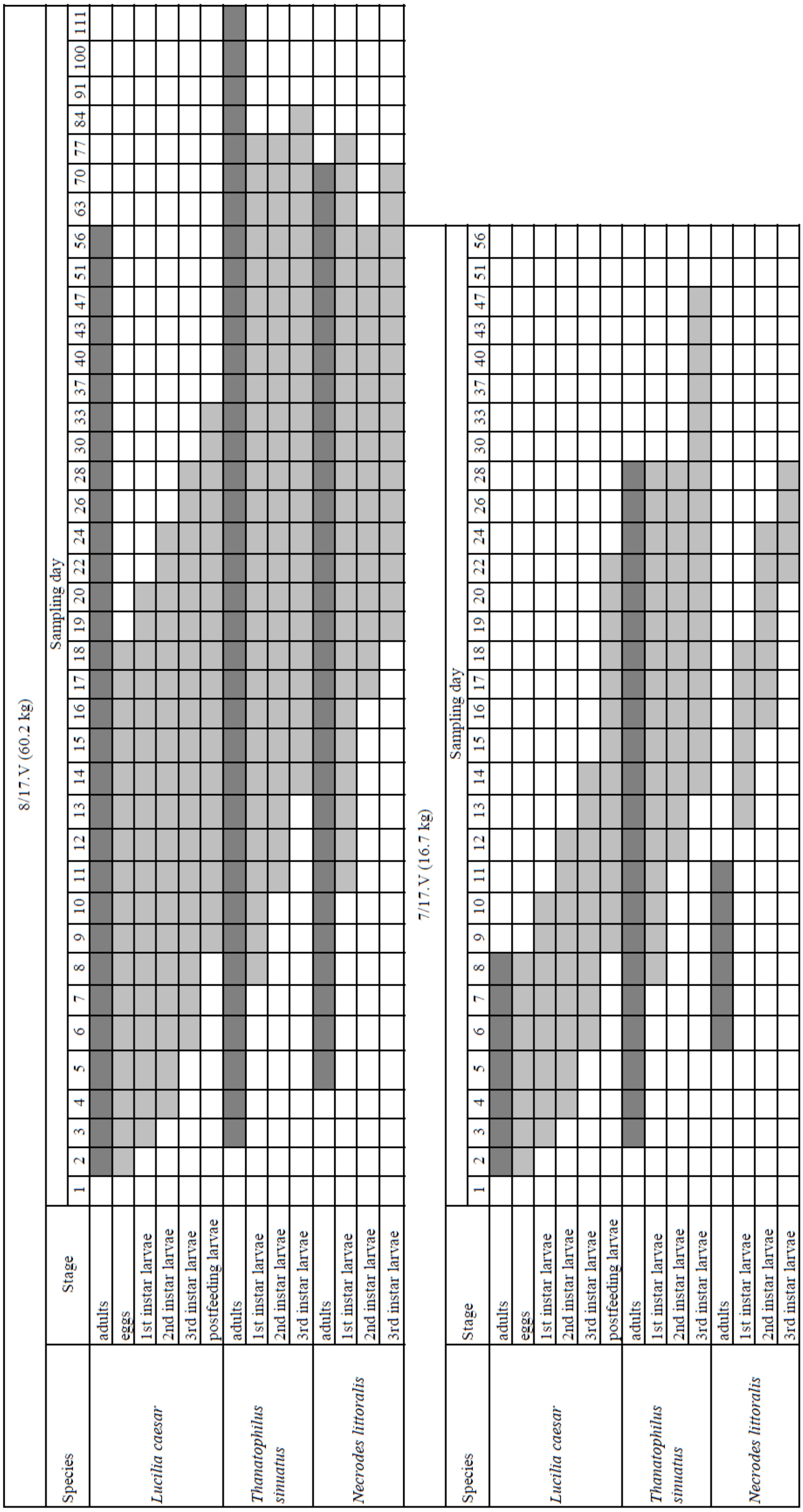

Fig. 2. Distribution of tested insect indicators over PMI as recorded on typical large (upper figure) and small (lower figure) pig cadaver.

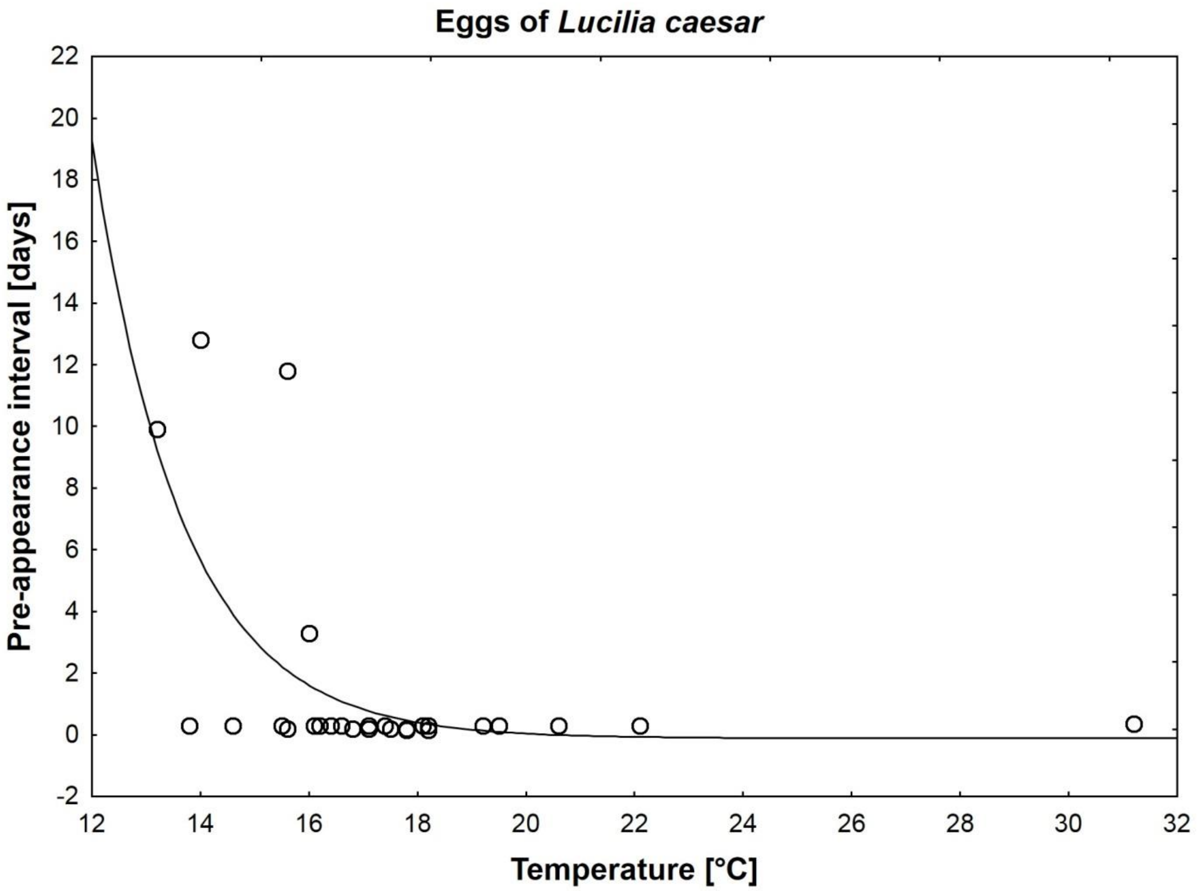

Eggs of *Lucilia caesar*
Pre-appearance interval [days]
Temperature [°C]
22
20
18
16
14
12
10
8
6
4
2
0
-2
12
14
16
18
20
22
24
26
28
30
32

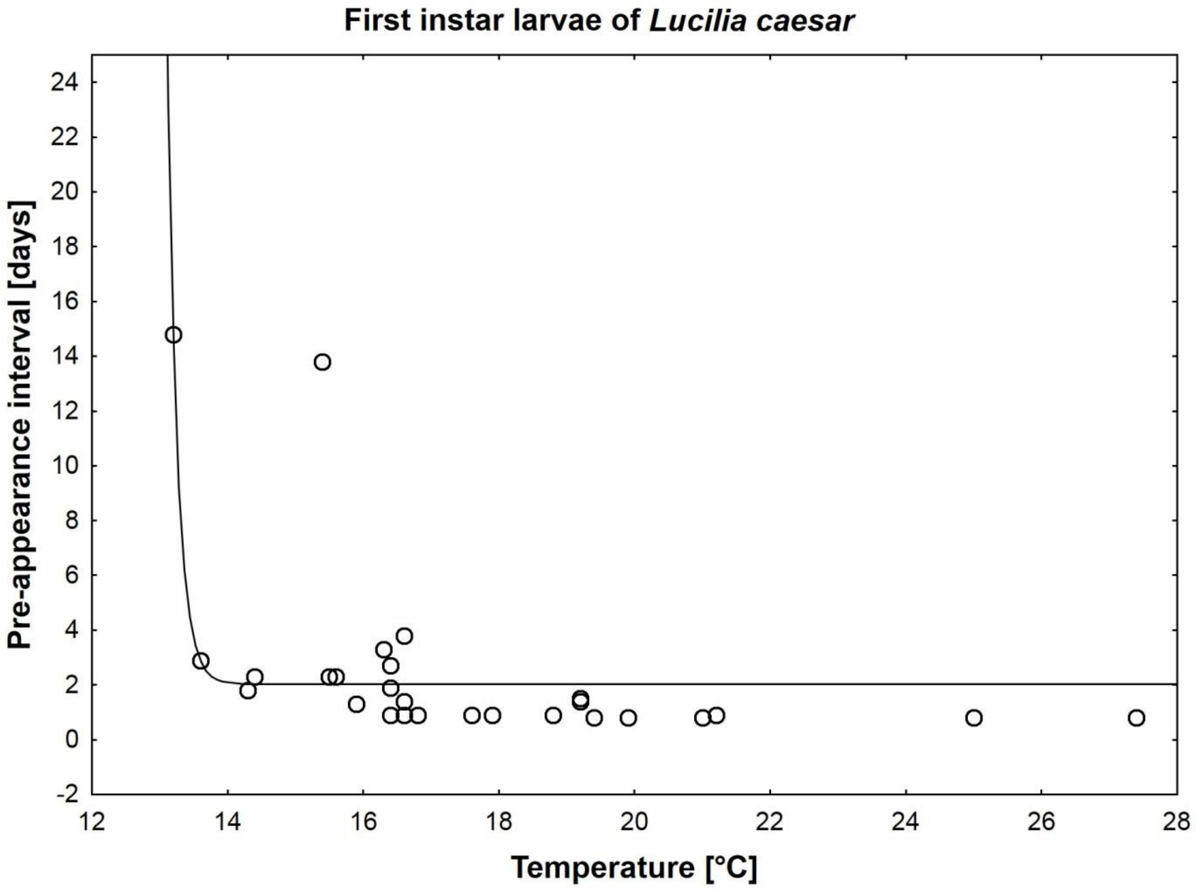

First instar larvae of *Lucilia caesar*
Pre-appearance interval [days]
Temperature [°C]
24
22
20
18
16
14
12
10
8
6
4
2
0
-2
12
14
16
18
20
22
24
26
28

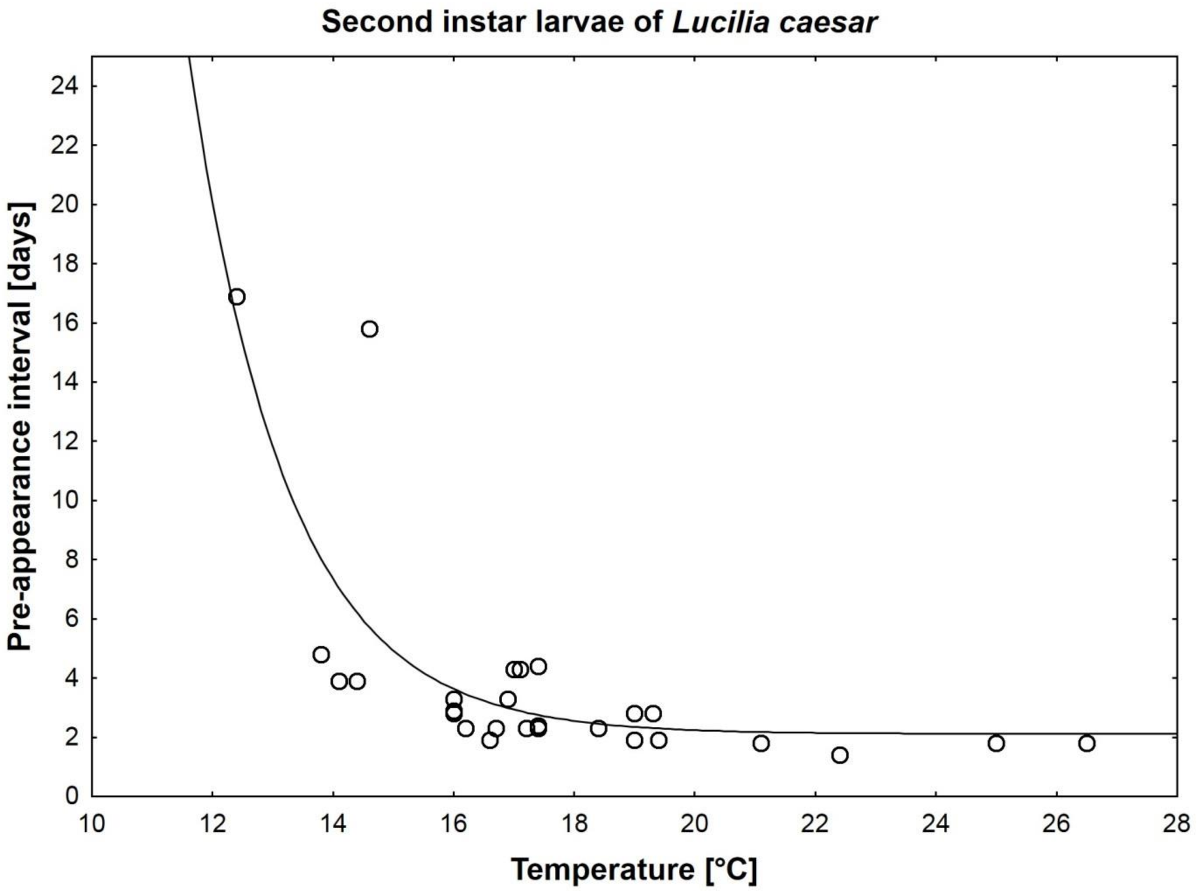
Second instar larvae of Lucilia caesar
Pre-appearance interval [days]
Temperature [°C]
0
2
4
6
8
10
12
14
16
18
20
22
24
10
12
14
16
18
20
22
24
26
28

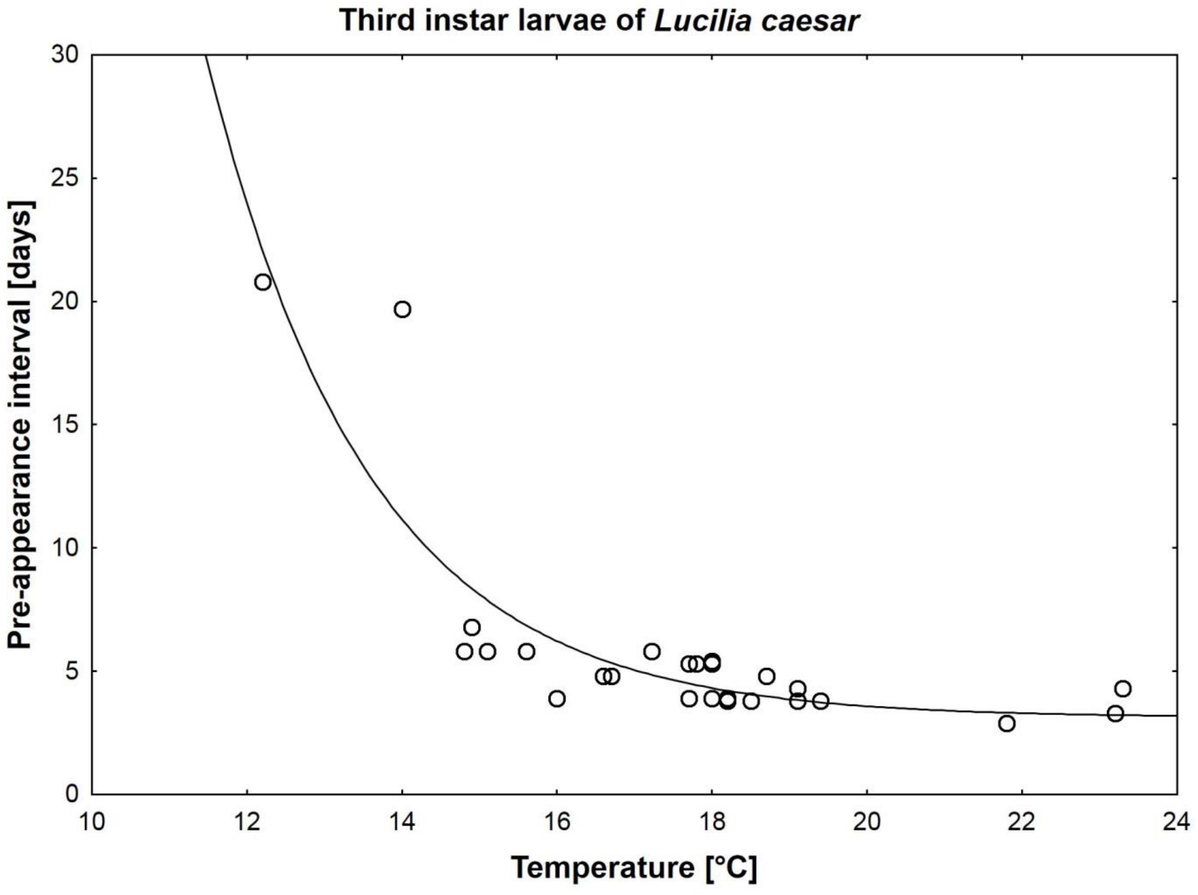
Third instar larvae of Lucilia caesar
Pre-appearance interval [days]
Temperature [°C]
0
5
10
15
20
25
30
10
12
14
16
18
20
22
24

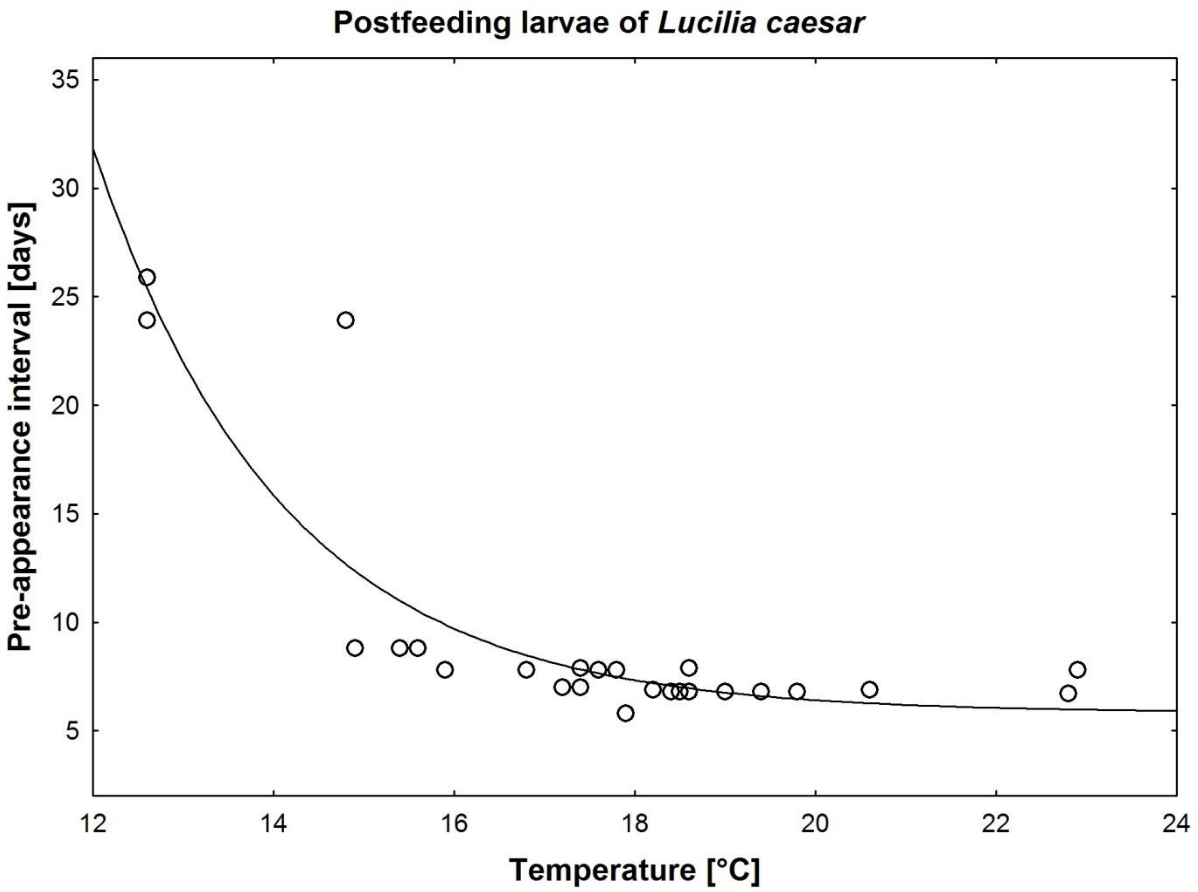

Fig. 3. The pre-appearance interval (PAI) of *Lucilia caesar* life stages plotted against ground level temperature averaged over PAI. Solid line – regression model of the relationship.

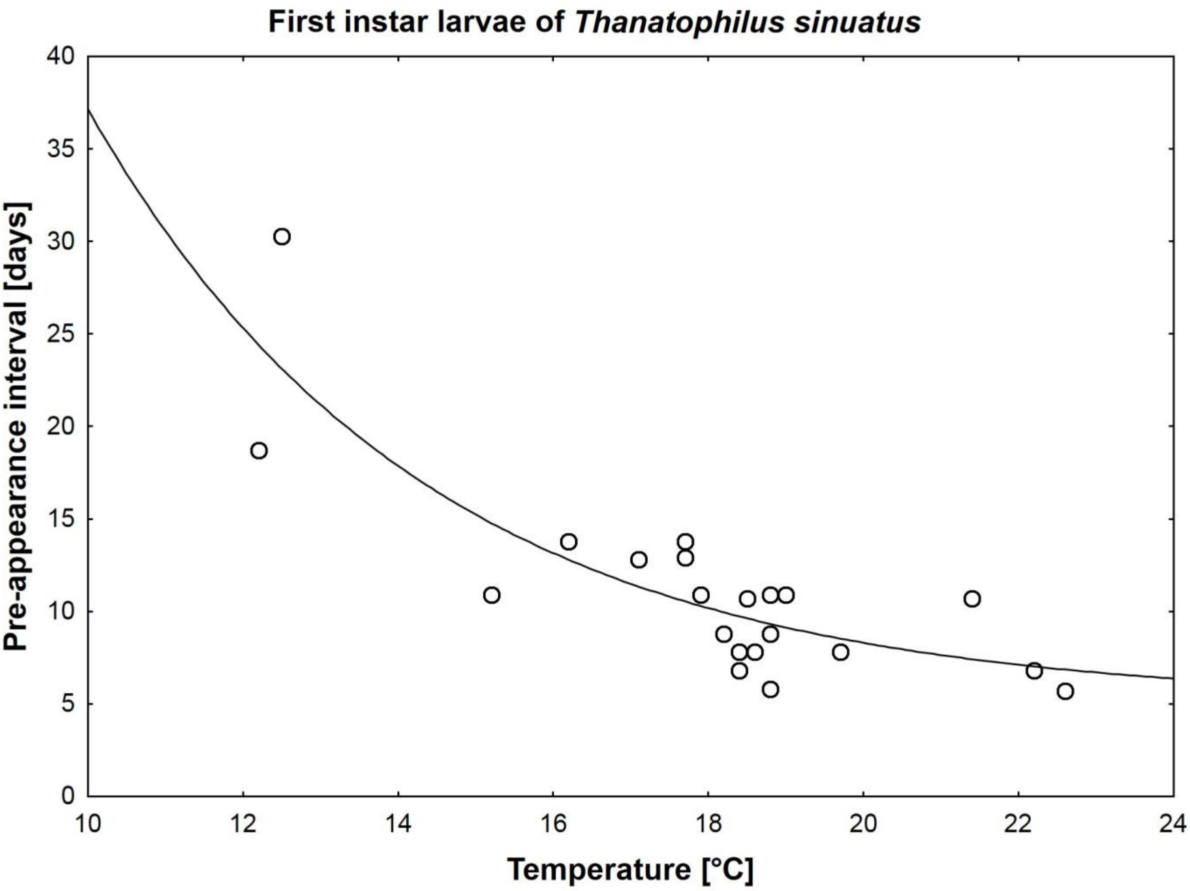
First instar larvae of *Thanatophilus sinuatus*
Pre-appearance interval [days]
0
5
10
15
20
25
30
35
40
10
12
14
16
18
20
22
24
Temperature [°C]

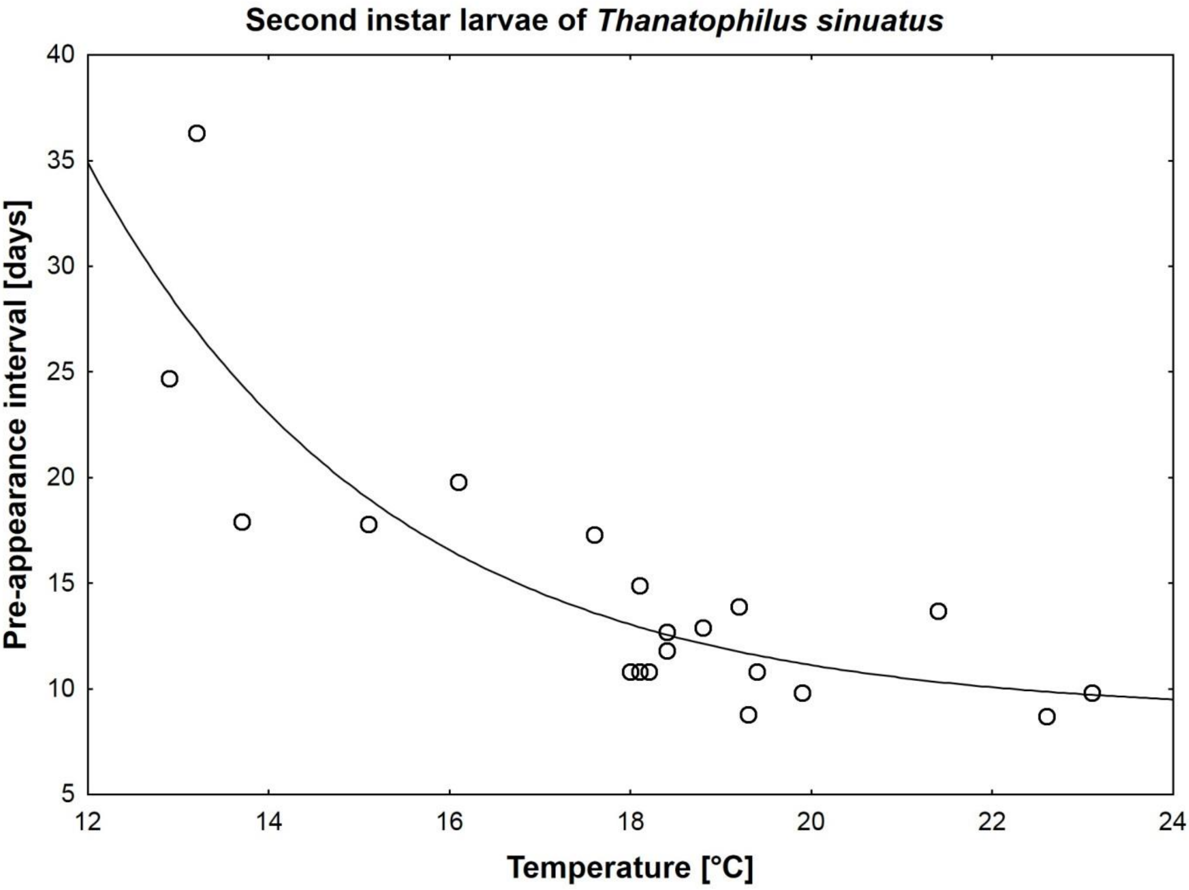
Second instar larvae of *Thanatophilus sinuatus*
Pre-appearance interval [days]
5
10
15
20
25
30
35
40
12
14
16
18
20
22
24
Temperature [°C]

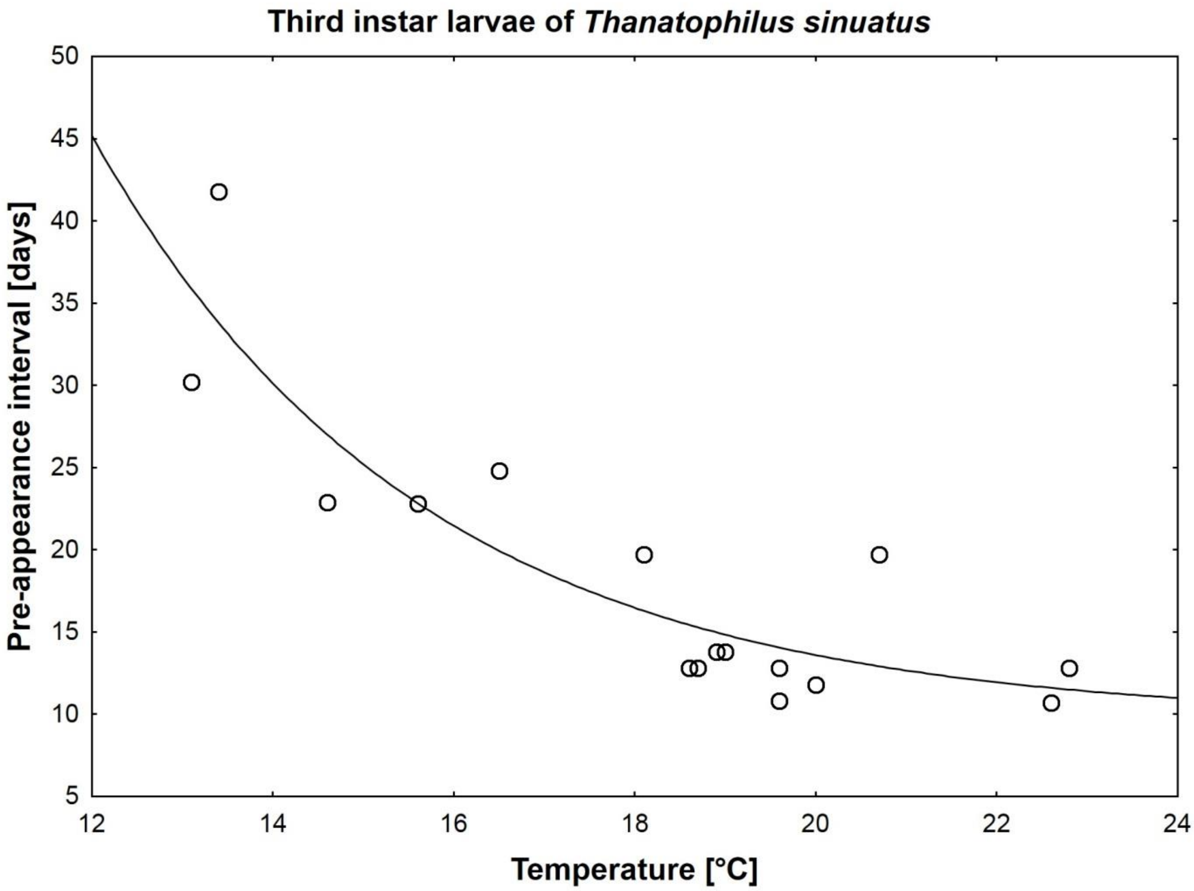

Fig. 4. The pre-appearance interval (PAI) of *Thanatophilus sinuatus* life stages plotted against ground level temperature averaged over PAI. Solid line – regression model of the relationship.

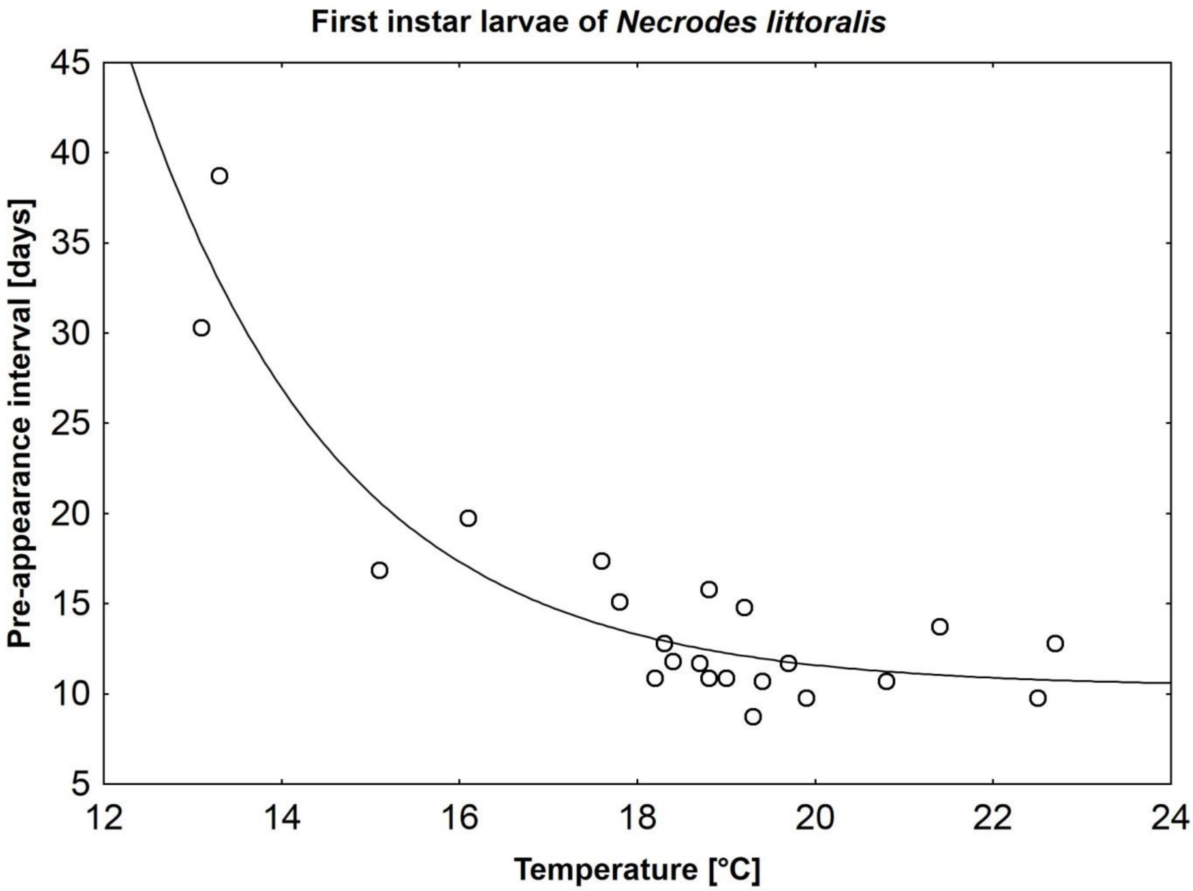
First instar larvae of Necrodes littoralis
Pre-appearance interval [days]
45
40
35
30
25
20
15
10
5
12
14
16
18
20
22
24
Temperature [°C]

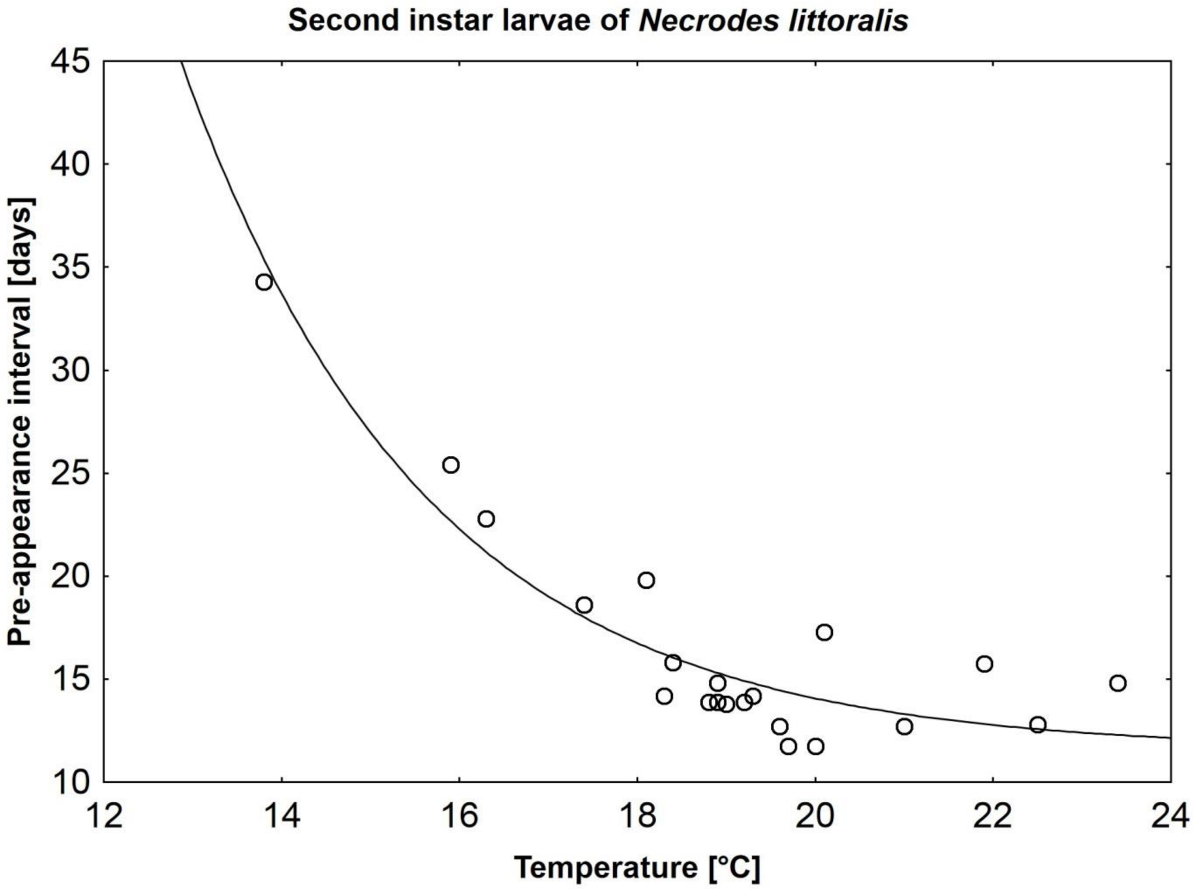
Second instar larvae of Necrodes littoralis
Pre-appearance interval [days]
45
40
35
30
25
20
15
10
12
14
16
18
20
22
24
Temperature [°C]

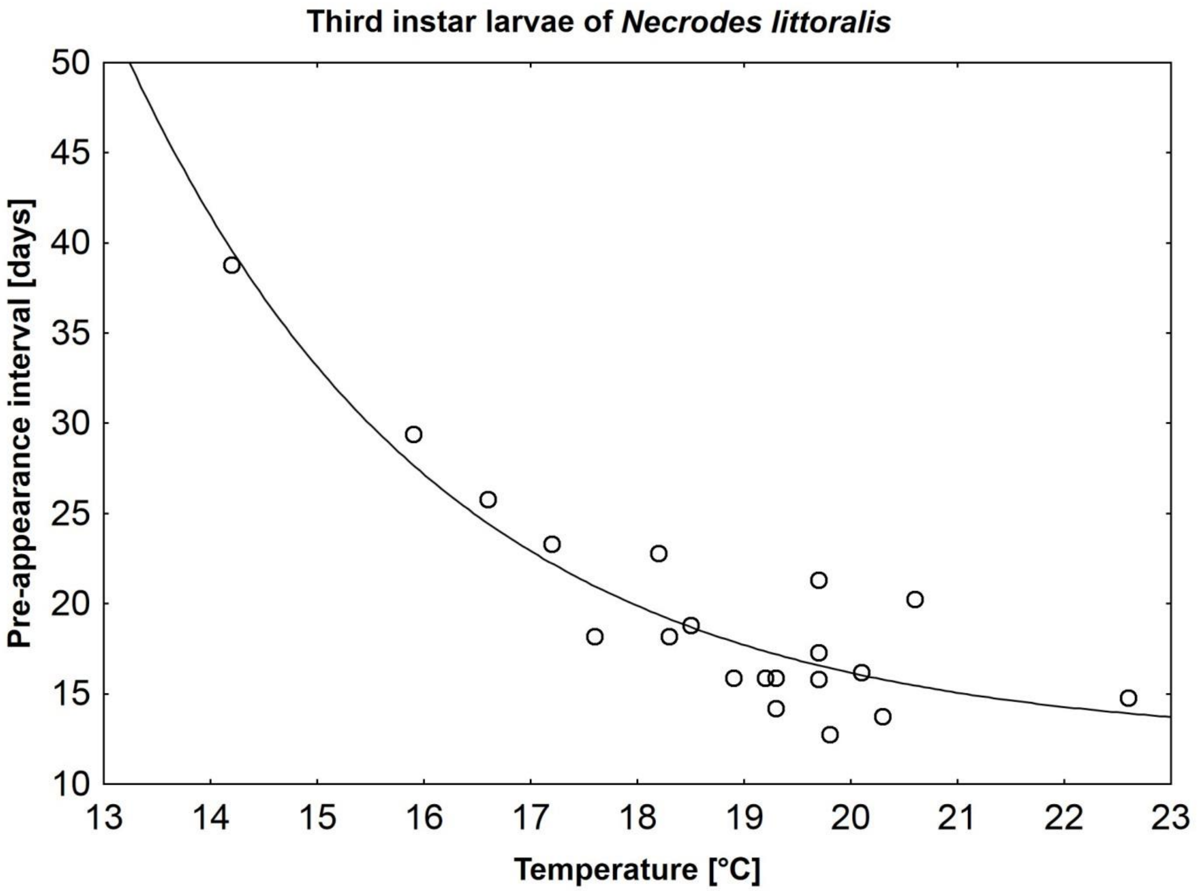

Fig. 5. The pre-appearance interval (PAI) of *Necrodes littoralis* life stages plotted against ground level temperature averaged over PAI. Solid line – regression model of the relationship.

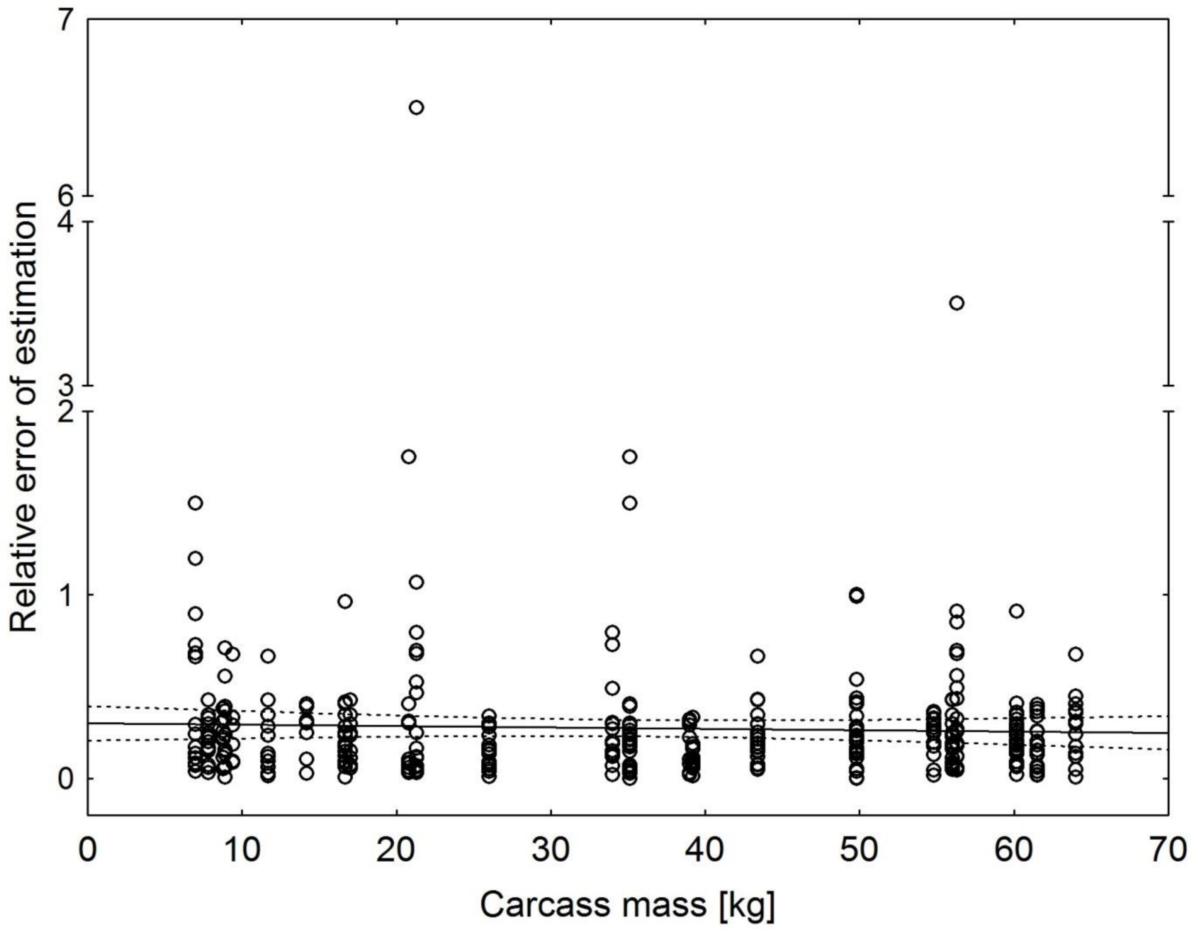

Fig. 6. Relative error of estimation (absolute difference between true and estimated PMI divided by true PMI) plotted against carcass mass. Solid line – regression model of the relationship.

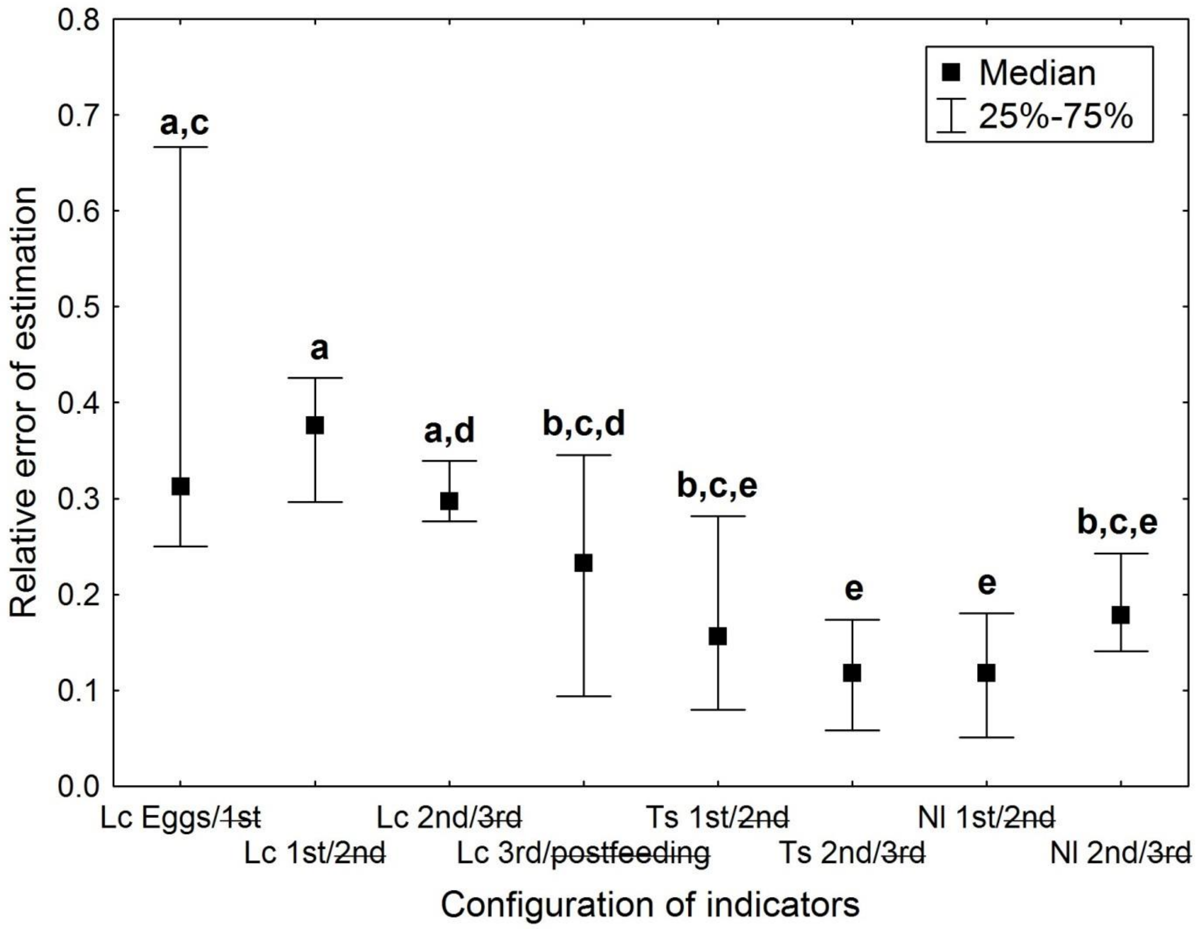

Fig. 7. Differences between configurations in the relative error of estimation (absolute difference between true and estimated PMI divided by true PMI). Different letters denote significant differences in pairwise comparisons ($P < 0.05$). Lc Eggs/~~1st~~ – presence of eggs and absence of 1st instar larvae of *Lucilia caesar*, Lc 1st/~~2nd~~ – presence of 1st instar larvae and absence of 2nd instar larvae of *Lucilia caesar*, Lc 2nd/~~3rd~~ – presence of 2nd instar larvae and absence of 3rd instar larvae of *Lucilia caesar*, Lc 3rd/~~postfeeding~~ – presence of 3rd instar larvae and absence of postfeeding larvae of *Lucilia caesar*, Ts 1st/~~2nd~~ – presence of 1st instar larvae and absence of 2nd instar larvae of *Thanatophilus sinuatus*, Ts 2nd/~~3rd~~ – presence of 2nd instar larvae and absence of 3rd instar larvae of *Thanatophilus sinuatus*, Nl 1st/~~2nd~~ – presence of 1st instar larvae and absence of 2nd instar larvae of *Necrodes littoralis*, Nl 2nd/~~3rd~~ – presence of 2nd instar larvae and absence of 3rd instar larvae of *Necrodes littoralis.*

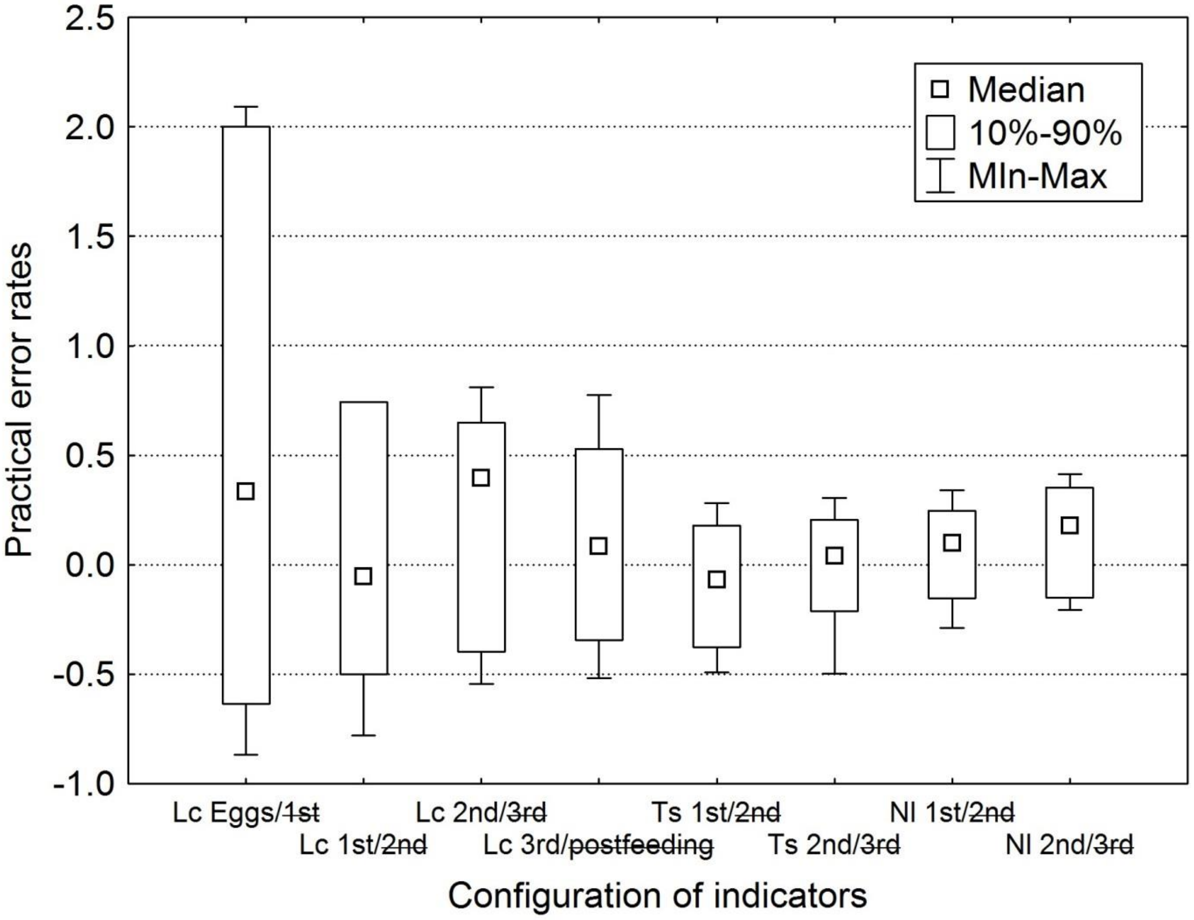

Fig. 8. Practical error rates (difference between true and estimated PMI divided by estimated PMI) for particular configurations. Lc Eggs/~~1st~~ – presence of eggs and absence of 1st instar larvae of *Lucilia caesar*, Lc 1st/~~2nd~~ – presence of 1st instar larvae and absence of 2nd instar larvae of *Lucilia caesar*, Lc 2nd/~~3rd~~ – presence of 2nd instar larvae and absence of 3rd instar larvae of *Lucilia caesar*, Lc 3rd/~~postfeeding~~ – presence of 3rd instar larvae and absence of postfeeding larvae of *Lucilia caesar*, Ts 1st/~~2nd~~ – presence of 1st instar larvae and absence of 2nd instar larvae of *Thanatophilus sinuatus*, Ts 2nd/~~3rd~~ – presence of 2nd instar larvae and absence of 3rd instar larvae of *Thanatophilus sinuatus*, Nl 1st/~~2nd~~ – presence of 1st instar larvae and absence of 2nd instar larvae of *Necrodes littoralis*, Nl 2nd/~~3rd~~ – presence of 2nd instar larvae and absence of 3rd instar larvae of *Necrodes littoralis.*
