# Supplementary material for: A general approach for postmortem interval based on uniformly distributed and interconnected qualitative indicators
Source: Int J Legal Med. 2017 Jan 4;131(3):877–84. doi: 10.1007/s00414-016-1520-3 (PMC5388707; doi:10.1007/s00414-016-1520-3)
Supplement: Supplementary file 2 — (PDF 214 kb) [file 414_2016_1520_MOESM2_ESM.pdf]

SUPPLEMENTARY TABLES FOR

"A general approach for postmortem interval based on uniformly distributed and interconnected qualitative indicators"

International Journal of Legal Medicine

Szymon Matuszewski

Laboratory of Criminalistics, Adam Mickiewicz University, Św. Marcin 90, 61-809 Poznań, Poland

szymmat@amu.edu.pl.

Content

Table 1. Definitive insect species for the rural habitats of Central Europe

Table 2. Temperature models for the pre-appearance interval (PAI) of developmental stages included in the analyses

Table 3. Average monthly pre-appearance interval (days) of the selected life stages of *Lucilia caesar*

Table 1. Definitive insect species for the rural (open and forest) habitats of Central Europe

Order	Family	Species
Diptera	Calliphoridae	*Calliphora vomitoria* Linnaeus, 1758
		Calliphora vicina Robineau-Desvoidy 1830
		Lucilia caesar (Linnaeus, 1758)
		Lucilia sericata (Meigen, 1826)
		Phormia regina (Meigen, 1826)
		Protophormia terraenovae (Robineau-Desvoidy, 1830)
	Fanniidae	*Fannia canicularis* (Linnaeus, 1761)
		Fannia manicata (Meigen, 1826)
		Fannia leucosticta (Meigen, 1838)
	Muscidae	*Hydrotaea dentipes* (Fabricius, 1805)
		Hydrotaea ignava (Harris, 1780)
		Hydrotaea pilipes Stein, 1903
		Hydrotaea similis Meade, 1887
	Piophilidae	*Stearibia nigriceps* (Meigen, 1826)
	Sarcophagidae	*Sarcophaga caerulescens* (Zetterstedt, 1838)
		Sarcophaga similis Meade, 1876
Coleoptera	Cleridae	*Necrobia rufipes* (DeGeer, 1775)
		Necrobia violacea (Linnaeus, 1758)
	Dermestidae	*Dermestes frischii* Kugelann, 1792
		Dermestes undulatus Brahm 1790
	Histeridae	*Margarinotus brunneus* (Fabricius, 1775)
		Saprinus semistriatus (Scriba, 1790)
	Nitidulidae	*Omosita colon* (Linnaeus, 1758)
	Silphidae	*Necrodes littoralis* (Linnaeus, 1758)
		Oiceoptoma thoracicum Linnaeus 1758
		Thanatophilus rugosus (Linnaeus, 1758)
		Thanatophilus sinuatus (Fabricius, 1775)
	Staphylinidae	*Creophilus maxillosus* (Linnaeus, 1758)
		Omalium rivulare (Paykull, 1789)
Hymenoptera	Pteromalidae	*Nasonia vitripennis* (Walker, 1836)

Table 2. Temperature models for the pre-appearance interval (PAI) of developmental stages included in the analyses

Species	Stage	Model*	r^2
Lucilia caesar	Eggs	$\mathrm{PAI} = (-0.114238) + e^{[(10.2233)+(-0.604897)\times\mathrm{Temperature}]}$	0.38
	1st instar larvae	$\mathrm{PAI} = (2.03038) + e^{[(91.2821)+(-6.72235)\times\mathrm{Temperature}]}$	0.49
	2nd instar larvae	$\mathrm{PAI} = (2.11191) + e^{[(10.3004)+(-0.617561)\times\mathrm{Temperature}]}$	0.61
	3rd instar larvae	$\mathrm{PAI} = (3.12195) + e^{[(8.7601)+(-0.476809)\times\mathrm{Temperature}]}$	0.78
	Post-feeding larvae	$\mathrm{PAI} = (5.83693) + e^{[(8.97272)+(-0.476234)\times\mathrm{Temperature}]}$	0.80
Thanatophilus sinuatus	1st instar larvae	$\mathrm{PAI} = (5.09098) + e^{[(5.76744)+(-0.23)\times\mathrm{Temperature}]}$	0.71
	2nd instar larvae	$\mathrm{PAI} = (8.81507) + e^{[(6.89735)+(-0.30304)\times\mathrm{Temperature}]}$	0.73
	3rd instar larvae	$\mathrm{PAI} = (9.70135) + e^{[(6.8821)+(-0.27611)\times\mathrm{Temperature}]}$	0.79
Necrodes littoralis	1st instar larvae	$\mathrm{PAI} = (10.3811) + e^{[(8.9028)+(-0.43531)\times\mathrm{Temperature}]}$	0.86
	2nd instar larvae	$\mathrm{PAI} = (11.5608) + e^{[(8.18248)+(-0.36313)\times\mathrm{Temperature}]}$	0.87
	3rd instar larvae	$\mathrm{PAI} = (12.3114) + e^{[(8.0968)+(-0.33734)\times\mathrm{Temperature}]}$	0.85

* $\mathrm{PAI} = c + e^{(b_0+b_1\times\mathrm{Temperature})}$, Temperature – ground level temperature averaged for the duration of PAI

Table 3. Average monthly pre-appearance interval (days) of the selected life stages of *Lucilia caesar*

Stage	Month				
	IV	VI	VII	VIII	IX
Eggs	11.6	0.2	0.3	0.3	0.3
1st instar larvae	14.3	1.0	1.2	0.8	2.2
